# Practices of Care in Participatory Design With Older Adults During the COVID-19 Pandemic: Digitally Mediated Study

**DOI:** 10.2196/45750

**Published:** 2023-07-17

**Authors:** Richard Paluch, Katerina Cerna, Dennis Kirschsieper, Claudia Müller

**Affiliations:** 1 IT for the Ageing Society Information Systems University of Siegen Siegen Germany; 2 Intelligent Systems and Digital Design School of Information Technology Halmstad University Halmstad Sweden

**Keywords:** older adults, care, Participatory Design, COVID-19 pandemic, digital health intervention, aging, health technology, digital media, gerontology, mobile phone

## Abstract

**Background:**

Participatory Design (PD), albeit an established approach in User-Centered Design, comes with specific challenges when working with older adults as research participants. Addressing these challenges relates to the reflection and negotiation of the positionalities of the researchers and research participants and includes various acts of giving and receiving help. During the COVID-19 pandemic, facets of positionalities and (mutual) care became particularly evident in qualitative and participatory research settings.

**Objective:**

The aim of this paper was to systematically analyze care practices of participatory (design) research, which are to different extents practices of the latter. Using a multiyear PD project with older people that had to take place remotely over many months, we specify different practices of care; how they relate to collaborative work in the design project; and represent foundational practices for sustainable, long-term co-design. Our research questions were “How can digitally-mediated PD work during COVID-19 and can we understand such digital PD as ‘care’?”

**Methods:**

Our data comes from the Joint Programming Initiative “More Years, Better Lives” (JPI MYBL), a European Union project that aims to promote digital literacy and technology appropriation among older adults in domestic settings. It targeted the cocreation, by older adults and university researchers, of a mobile demo kit website with cocreated resources, aimed at improving the understanding of use options of digital tools. Through a series of workshops, a range of current IT products was explored by a group of 21 older adults, which served as the basis for joint cocreative work on generating design ideas and prototypes. We reflect on the PD process and examine how the actors enact and manifest care.

**Results:**

The use of digital technology allowed the participatory project to continue during the COVID-19 pandemic and accentuated the digital skills of older adults and the improvement of digital literacy as part of “care.” We provide empirically based evidence of PD with older adults developing digital literacy and *sensitizing concepts*, based on the notion of care by Tronto for differentiating aspects and processes of care. The data suggest that it is not enough to focus solely on the technologies and how they are used; it is also necessary to focus on the social structures in which help is available and in which technologies offer opportunities to do care work.

**Conclusions:**

We document that the cocreation of different digital media tools can be used to provide a community with mutual care. Our study demonstrates how research participants effectively enact different forms of care and how such “care” is a necessary basis for a genuinely participatory approach, which became especially meaningful as a form of support during COVID-19. We reflect on how notions of “care” and “caring” that were central to the pandemic response are also central to PD.

## Introduction

### Background

The COVID-19 pandemic prompted many societal changes including who we care for and how. In this paper, we examine the care dynamics between socioinformatics researchers and older research participants in a Participatory Design (PD) project in a German city. Through the lens of care as a process according to Tronto [1,2], we analyzed the project and the way it dealt with COVID-19 and its consequences [3].

This paper builds on and reinforces the lessons from previous research on helping older people acquire technical skills, conducted both before and during the pandemic (for a review, refer to the paper by Ahmad et al [4]). Although these studies have continued to emphasize the importance of internet technology for older adults [5,6], they also record a lack of confidence in the use of technology and the consequent resistance to learning among older users [7]. Most studies have documented the need to enhance instructional design use in teaching older adult learners. In general, Lin and Chang [8] suggest that strategies for teaching technology to older users should address a range of issues including overcoming physical barriers, developing and presenting a structured but flexible curriculum, and encouraging mutual engagement between learners and teachers.

For example, there is considerable literature that examines the use of tablet technology [9,10]. Other studies consider the process of learning more generally from a range of perspectives [11]. Chiu et al [12] used interviews and observational studies to develop a multiple-case research method concerned with understanding aspects of older adult learning and the corresponding teaching strategies at senior learning centers, suggesting that successful instructors developed a range of different teaching strategies for older learners.

Chaudhry et al [10] used workshops and observational data to identify particular physical and intellectual challenges with the use of tablet computers by older users, including the use of a touchscreen and issues of trust concerning sharing personal information suggesting that “[p]airing older adults with mentors and encouraging independent learning were the key teaching strategies” [10]. Similarly, the study “Social Support and ‘Playing Around’: An Examination of How Older Adults Acquire Digital Literacy With Tablet Computers” by Tsai et al [9] used interview data to emphasize the importance of “messing around” and social support in developing tablet use. More recently, and with specific reference to the impact of the COVID-19 pandemic, Bangert et al [13] pointed to the intrinsic motivation of adult learners to take web-based courses being held back by the lack of confidence as well as access and the consequent need to modify curriculum and teaching and learning strategies. These themes are also emphasized in the systematic literature review by Ahmad et al [4].

The research project at hand, ACCESS [14], which aimed to design tools to help older adults access the digital world more easily, adopted a PD approach, which is less about a particular data collection or analysis techniques than it is concerned with an attitude or set of values that shape the design project [15,16]. These values were originally focused on Scandinavian concerns with social democracy, participation, and workplace empowerment but also included ideas about the situated character of design, designing for specific settings, developing common understandings, mutual learning, equality, and appreciation [17]. The approach has evolved over time but has increasingly emphasized values [18] and value-based strategies of engagement that allow meaning and decision-making to emerge in varied and occasionally contentious private and public contexts [19,20].

The value system emerging from the Scandinavian tradition of PD consists of a set of general, stable values shared by a community of researchers and design practitioners. However, the advent of COVID-19 in particular has presented new challenges and opportunities for PD, especially regarding ideas about engagement and inclusion [21] within an overall social and political approach that has notably focused on the issue of care, care for oneself, and care for others [1,2]. It is this set of values that has predominantly impacted and motivated our research.

The project reported here started before the COVID-19 pandemic began. When the pandemic began, it became clear that though it was impossible to continue the research in its current on-site format (because it was unsafe for the participants and irresponsible to endanger them), it would be wrong to stop the research completely because it was a promising way for the older adults to be in contact and engaged (see also the study by Lebrasseur et al [22]). The use of digital technology allowed the participatory project to continue, as well as draw attention to the digital skills of older adults and ways to improve their digital literacy [23,24].

### Theory

We focus on Tronto’s [1,2] notion of care because we believe it can be productively related to our assumption that care practices are pivotal elements of PD or participatory research processes and Tronto’s conceptualization is helpful in making them more visible and usable for planning and conducting PD work.

Tronto [1] developed a 4-phase model of care that differentiates care into “caring about, taking care of, care-giving, and care-receiving.” “Caring about,” here labeled as the first phase, refers to a recognized need that has to be met. The recognition can take place at either the individual or the societal level. The second phase of the caring process is “taking care of,” which refers to taking responsibility for the need and determining what needs to be done. “Care-giving” is described as a direct action that takes place physically through contact with a recipient. “Care-receiving” refers to how a recipient responds to a care practice. Tronto [1] relates this 4-phase model to aspects of power theory. Thus, the phase of “caring about” and “taking care of” could be connected with power and masculinity [25]. For Thelen [25], for example, the aspect of “taking care of” would become clear in a male breadwinner conception. According to this view, the man would be the one who takes care of the family by means of his gainful employment because this would enable him to dispose of the money. The woman in this worldview would be able to take care of the children or other family members (“care-giving”) in the next step. This reveals further differentiation regarding the social position of the carers and the power structures of patriarchal societies that foster such conceptions [26]. Moreover, care does not have to be thought of in concrete contexts of action or tied to specific types of relationships.

In a later publication, Tronto [2] added a fifth phase: “caring with.” The fifth phase is about caring together, and thus, about the stabilization or permanence of care actions on a societal level.

Several researchers have referred to Tronto [1,2]. For example, Krüger et al [27] address the relevance of care in participatory technology development studies with people from migrant backgrounds. They highlight that it is relevant to differentiate how participation is linked to care and power and how PD can also mediate hierarchical care relationships. Groot et al [28] make a similar statement, explicitly referring to the health care context. We considered these aspects in the course of our study and used them to inform our analysis. For example, regarding power and care, this meant reflecting on who is in a privileged position or what dependencies exist. Furthermore, it meant having the possibility to reflect on such aspects and realize, as well as discuss, which interests are addressed and how, and which resources can be used [29,30].

Owing to the COVID-19 pandemic, the ACCESS research project had to move from on-site meetings to web meetings, and the conditions for care practices changed because a considerable amount of caring relies on direct physicalities, such as handing another person a drink or touching their hand, but of course, none of that is possible in a Zoom meeting (Zoom Video Communications, Inc) [31]. To argue that a Zoom meeting can nevertheless be a place of care, we take up the distinction between “space” and “place” according to Harrison and Dourish [32]. Here, “space” refers to a space of possibilities and “place” refers to the concrete practices that give this space a certain meaning. For example, a space in a city becomes a marketplace when goods are exchanged there, whereas the same space can become a party place when people have a party there. Although a Zoom meeting is not a physical place, it is still a space with possibilities for different uses. For example, if it is used for caring conversations, it becomes a place of care. Moreover, with this distinction, according to Harrison and Dourish [32], the interrelation between care and PD can be better conceptualized, as we see that different practices, such as caring for each other and working together on a design project can overlap in the same space of possibility, such as a Zoom meeting.

### Objective

The project followed a socioinformatics research paradigm targeting societal inclusion and empowerment through technical artifacts as a design goal and premise of the research process [33]. This understanding of design practice includes the task of establishing and maintaining long-term, everyday-life–oriented, and trust-based research cooperation with target group representatives (ie, study participants). Our primary concern in this context was how care can be understood as a broad concept and process and how it can be enacted as the basis for participatory approaches [34]. Our research questions were “How can digitally-mediated PD work during COVID-19 and can we understand such digital PD as ‘care’?” Hence, PD is understood as a way to create sociotechnical structures that are beneficial for care and in which people can support each other, thereby highlighting and presenting important “implications for design” for both the present and future of pandemic technologies.

We reflect on our PD process and the ways in which care situations and relationships changed or developed anew. This type of socioinformatics research involves permanent reflection loops on the methods and means aiming at equal cooperation and collaboration and the limiting and hindering factors stemming from the sociocultural contexts of both the (older) participants and the university researchers [35]. Thus, it is important to have a caring participatory approach in which research participants are not objectified or seen solely as data sources. PD per se includes acts of care. These acts or practices of care have not been so far made visible as important elements of the PD process and project. With Tronto’s [1,2] conceptualization, we can capture them better and reflect on how to improve PD with older adults and in remote settings.

## Methods

### Study Design

This paper is based on the European research project, ACCESS, where PD took place at different levels [36]. The project involved older adults unfamiliar with digital technologies but who had a strong interest in cooperating with the university that many of the participants had already worked with. The focus was initially on enabling for co-design and slowly approaching the collaborative project task of co-designing appropriation support tools [37,38]. As some members of the author team (DK and CM) are involved in other research projects in which participatory research was continued on the web owing to the pandemic, reflections and conceptual considerations from these projects have influenced this study. This is a subproject of the Collaborative Research Center 1187 and Swiss National Science Foundation–Swiss National Research Programme 74 CareComLabs, which address caring communities and, in contrast to ACCESS, focus more clearly and independently of the pandemic on the topic of giving and receiving care and help.

Methodologically, in this paper, we are guided by PD [34] and thematic analysis [39].

Our work is based on a participatory research design approach, integrating the participants directly into the research process from the beginning. PD is a methodological approach based on enabling the participation of all relevant stakeholders in a design project [34]. There are different forms of participation and degrees of involvement. We did this by understanding the people who helped shape the project as research participants. Older people are understood here as experts in their everyday lives [40]. Our specific approach aims to understand people’s living contexts and how they think about or locate digital tools in their homes and surroundings and their sense-making processes. A specific aspect of ACCESS was the coupling of cocreating ideas for technology design with strategies for sustainable appropriation and learning support for older learners.

### Research Project ACCESS

ACCESS is an interdisciplinary and multinational project with 5 European partners that was conducted from 2019 to 2021. ACCESS aimed to promote digital literacy and appropriation of digital tools by older adults in a low-threshold and everyday-oriented way. ACCESS’s particular research goal was to pursue the design of age-friendly technologies while coreflecting and co-designing care places.

We wanted to create a learning environment where, through PD, older adults would become capable of developing their self-directed learning. The national subproject whose team’s work is reported in this paper is based in a German town and focuses on the role of PD in this process and on exploring the measures for including older adults in participatory and co-design processes as a way to increase their digital literacy. We aimed at the cocreation of a mobile demo kit. The mobile demo kit is *a website* with a collection of resources cocreated with the older participants, aiming to improve digital literacy and everyday appropriation of digital media with and for older people in their homes [41].

### Research Participants

In the ACCESS project, 21 older persons were involved, with an age range of 65-80 years. The people had different levels of digital literacy; some can be described as beginners and others as experts, as well as experienced with participatory projects conducted with universities. They also gave different motivations for participating in the project. It was sometimes mentioned that they wanted to socialize with younger people, learn about university work, teach other people how to use digital tools, or learn how to use these technologies themselves. Participatory workshops were held to collect the data. The research team comprised the second and last authors (KC and CM) of this paper. In addition, the students from our university assisted with the workshop and were paid for their work. The students knew some of the participants and were thus familiar with them. The workshop plan was that some technologies would be used by older adults to support them. This included smart speakers, smartwatches, and self-tracking devices, as well as everyday technological artifacts such as cell phones and tablets. The focus of the first workshop was to familiarize older adults with digital tools. This was a preparatory phase. Through exposure to these technologies, further technical skills could be developed. If a person learns how to use cell phones or tablets, in the second step, they can be taught to communicate via instant messengers, use tools for videoconferencing, or understand how tracking apps work.

### Ethical Considerations

Ethical Committee review and approval were waived for this study as older adults were invited to become participants who were fully compliant. Due to a long-standing cooperation arrangement with members of a local senior association they were invited by the researchers for a volunteer participation. All participants engaged voluntarily in the participatory research activities and they had the possibility to opt out at any time. Informed consent covered sensitive and private information. The authors were guided by the Association for Computing Machinery (ACM) code of ethics.

### First Impacts of COVID-19

As the pandemic progressed in the beginning of 2020, making it impossible to meet on site, it was quickly decided, together with the older adults, to continue conducting this type of research. For example, interviews were conducted with older persons to learn about their concerns and needs. For the meetings during the lockdown, different videoconferencing tools that would best fit our situation were explored (eg, Skype [Skype Technologies], Jitsi [8x8 Inc], and Zoom [Zoom Video Communications]). Finally, we chose Zoom because it proved to be the most robust when it came to unstable connections and because the university provided us with a license that allowed us to have an unlimited number of meetings. The data presented in this paper are based on these web meetings and show how PD changed through COVID-19 and how the care concept gained importance.

### Workshop Series

#### Overview

This study was conducted through a series of workshops. Altogether, we organized 29 workshops (5 on-site and 24 on the web). The average duration of each session was 111 minutes, and the average number of participants was 7. Almost each web session was video recorded and transcribed. The first web session was not recorded because of technical issues. We documented this session with field notes, screenshots, and a short (90 seconds) video. Each in-person session was audio recorded and accompanied by field notes and photographs, when suitable. Each session was followed by a short briefing session, in which challenges and relevant moments were discussed. These sessions were also documented using field notes and further used for our analysis. We used these data for the qualitative analysis [42].

**Figure 1:**
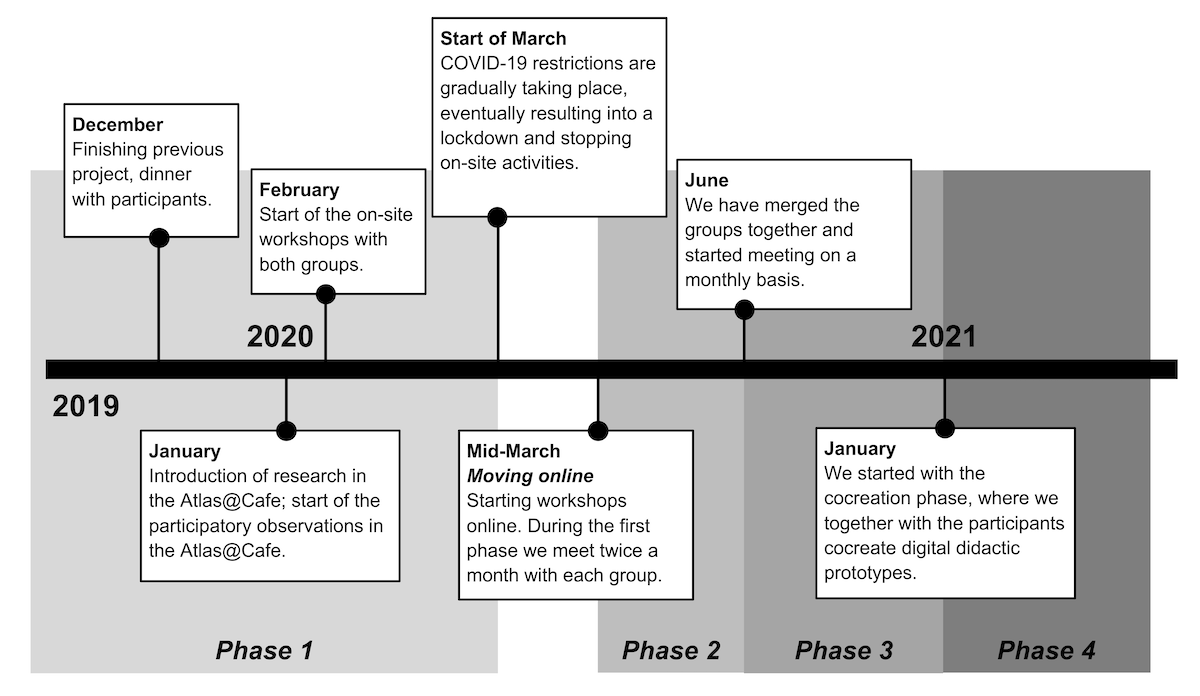
Timeline of the research work (adapted from Cerna et al [42], which is published under Creative Commons Attribution 4.0 International License [CC BY 43]).

The following distinction aims to provide a better understanding of what the workshops aimed at the phases as described in the following sections (see also Figure 1).

#### Phase 1: On-Site Workshops and Field Observations

During this phase, we organized on-site workshops in 2 parallel groups. We started by getting to know each other and setting up the infrastructure for our collaboration, such as instant messenger tools. The main aim of this phase was to establish a mutual social and learning ground for workshops.

#### Phase 2: Transition to the Web

The pandemic started and we moved to the web. We were meeting with each of the groups once every 2 weeks. The main goal was to teach the participants how to navigate Zoom and establish forms of cooperation.

#### Phase 3: Learning in 1 Group

We merged the 2 groups into 1 and started to meet once a month. We began to explore further digital tools and how to explore them in a web-based setting.

#### Phase 4: Co-Design

On the basis of the mutually developed ground through the previous phases, we started to co-design artifacts together, including creating instructions for how to use a robotic vacuum (iRobot), redesigning a bank flier so that it better fits the needs of the older participants, and creating instructions on how to get a subscription for newspapers on the web. All artifacts aimed to be included in the demo kit [41].

More details about the empirical work can be found in our previous study [42].

### Data Analysis

We analyzed our data using an approach informed by thematic analysis [39]. Thematic analysis aims to identify patterns of meaning or themes in qualitative data sets. Here, coding is the process of detecting relevant aspects of data that show shared meanings and are of particular importance to the research question. Themes are found when relevant aspects are related to each other and differences and similarities are identified. In this study, we specifically focused on particular themes that emerged regarding acts or practices of care. We used care as a lens (as a form of deductive element) for our inductive analysis (eg, by reading the video transcripts, study diaries, and collected field notes). With each subsequent analysis, the care themes were refined, combined, or newly formed. In this analyst-driven approach, it was important for us to accurately interpret the data [39].

## Results

### Caring Spouse Hindering Researchers’ Instructions

Conducting web-based PD was not part of the original plan for the ACCESS project. Although other projects stopped their work or minimized it, for our project, we decided to continue working with our participants in a way that created new opportunities and addressed some of the issues that older adults experienced during the COVID-19 lockdowns.

For example, several of our participants were dependent on their family members to help them join the web workshops:

One of the participants often expresses that he is thankful for the workshops and thinks he needs them. During this session, he logged in with his son, through a phone of one of them. At the beginning the son said that he himself was not hundred percent sure how to use Skype. They left after some time, because the phone was getting too warm.

Field note from the workshop

This support can be viewed as “care,” as a way to enable participation in a context that is important to them. However, this caring relationship sometimes interfered with our approach. During one of the first workshops, participant P had trouble logging into Zoom. One of the researchers supporting the workshops called her and tried to provide instructions on how to proceed with Zoom. This phone call took a very long time because her husband was sitting next to her and trying to provide her with instructions simultaneously. In this case, the caring relationship between the partners disrupted our efforts to enable participation in the design space for one of them.

This is an aspect that is similar to when research takes place in the homes of people [44]. Through the scientific study, we also had a window into the participant’s private sphere, which allowed other people to participate in the care place. This represented a creative way of dealing with a situation that we were not prepared for. In future situations, we should respond better when relatives join in.

### Design Space as a Window

The following example illustrates how providing care to a friend meant hindering participation for others within the design space. Participant O was one of the frequent attendees of our web workshops. During one of the web workshops, participant V (who we knew from previous collaborations) suddenly emerged in the house of participant O. She came there to ask him for help with her smartphone. Participant V noted the following in her study diary:

They could help me only over the phone, but that did not work for me. Then I had asked my neighbor and he suspected that I had made a mistake when installing the new version. So I still have a lot to learn.

Notes from the study diary of participant V

As participant O did not know how to mute his microphone, their “caring conversation” interfered with our workshop and stopped it from proceeding in a smooth way (because the shared audio channel got “blocked” by their conversation).

This example is similar to the one above but differs in that one person actively took the initiative to obtain help from another participant. It also shows how intertwined the computer-generated space and the analog space are and how switching from one to the other can change the interaction situation. It further showed that helping is an expected practice in this place of care that we opened. However, the participants did not assume that only the researchers from the university should support others but that they could also accept the role of a helper themselves. Thus, it represented a negotiation process in our design setting and showed how different expectations can motivate older adults to help each other.

### Instructors: Caring for the Cause and the People

Some of the participants who took part in our project were volunteer instructors in a local computer club for older adults run by their peers. As they were skilled in the daily use of digital technology, they did not need additional digital literacy support. Despite this, they participated in almost all of our workshops. Their participation was an expression of 2 elements.

First, this was a matter of care for the cause, the digital literacy of older people and ways of support are issues that have to be handled on a personal, local, and communal level. For example, during the first introduction to our project, instructor Q expressed she will participate because she wants to “keep an eye on us [the researchers].”

Second, this was also a matter of helping the participants during the web workshops. The workshops were very demanding, and sometimes, when the participants had troubles, the instructors stepped in and provided them with support.

Third, it was a matter of supporting us researchers in better understanding the issues of the older participants. During one of the web sessions, we ran into trouble again with supporting the research participants in reaching the digital meeting room. One of the researchers tried to provide instructions, but the participant still struggled. At that moment, instructor Y explained that the reason the support was not working was because the researchers were using a term (“digital ecologies”) that was confusing for older adults. This was a surprise because we used this term for almost a year and no one complained. It shows that any “scientific” language has to be adapted to the particular life circumstances of the research participants and constantly monitored and that this is an aspect of “care.”

## Discussion

### Principal Findings

Through its participatory approach, the activities of project ACCESS opened a design space where it was possible to not only (1) engage with older adults and foster their learning to use digital tools but also (2) understand their daily issues that are heavily impacted by the COVID-19 restrictions with or without relationship to technology. Both aspects (engaging and understanding) are mutually constitutive of each other and, in turn, foster each other [31]. Enabling older adults to participate in contexts where they can learn using technology was essential as it helped them continue engaging in interpersonal relationships even during COVID-19 and despite the restrictions. Enabling their participation was essential but not sufficient; what made the difference were the care practices. Furthermore, the sessions were designed to further develop the demo kit website [41] and empower individuals to be more autonomous in future PD workshops (eg, being able to log on to web meetings and participate in conversations without the help of others).

Participatory research and design are broad fields with different ways to conduct research [45]. We refer to the concept of care as our contribution to this discourse [46]. Participatory approaches to research have long claimed to create or provide researchers and participants with opportunities to gain knowledge, empowerment, and the challenging or rethinking of power structures, as well as the possibility of gaining or developing knowledge [47]. Instances when participation in a research project has (perhaps unintentionally) contributed to various forms of well-being or care have perhaps been less well-documented [48].

In general, there is a need for participatory empirical studies to gain a better understanding of the actual practices and attitudes of older persons regarding the use of assistive digital technologies [49]. Our interest lies in addressing the different phases of care to make the invisible aspects of learning and relationship building in PD visible.

### Practices of Care in PD With Older Adults

Here we present some reflections on the notion of PD through our theoretical care lens of “design as care” as informed by Tronto [1,2].

First, there was the necessity to recognize the need for care: “Caring about: recognition of care needs.” This is the first necessary step to recognize a care need. However, how care should be recognized and by whom is a situated range of choices rather than a predetermined action. It is a widely established fact that older adults’ needs are not reflected and well matched in the currently accessible tools [50,51]. However, one of the main reasons why our participants decided to participate in our project was that they wanted to learn how to use digital tools. They recognized themselves as someone who needed help, in other words, in need of care (eg, the information in the study diary of participant V). However, this does not mean that every older adult will perceive themselves as someone who needs it—both the care and digital tools. In addition, care and technical artifacts are also 2 separate issues that can be thought of as completely independent of each other. There are also PD workshops in which no technical artifacts are used. However, the videoconferencing tools in our workshop were necessary for safe exchanges to take place during the COVID-19 lockdowns.

Thus, an important part of our PD process was to show the older adults the current technological developments and through mutual learning provide them with an understanding of the tools that fit their needs (and which are irrelevant for them) [27].

The next phase of care is “Taking care of: acceptance and allocation of responsibility (who attends to care needs?).” The participants were provided with care related to their digital tool needs through various modes within the PD process. First, the entire PD process was designed by us researchers with the broad assumption of caring for older adults. None of the researchers necessarily identified as an older adult. However, through previous empirical projects (eg, Collaborative Research Center 1187 and Swiss National Science Foundation- Swiss National Research Programme 74 CareComLabs), we identified the existing needs of the older adults.

Second, instructors from the local computer club also participated in our workshops. They decided that they wanted to participate in the process and then also took a more active role. We saw their active role as an important process in community development. Their participation was a way to care for their peers and to support us.

Third, the actual “care-giving” took place where the above-identified people took charge in multiple ways. The broader ideas of who should care for the older adults were translated into actual practical actions during the workshops, when we helped them to figure out their issues with their devices or when we moved the whole series of workshops to the web so that we might continue collaborating. The instructors also provided care during the workshops. Sometimes, it was necessary to build “bridges” between the researchers and older adults so that they could make sense of the situations in the workshops. For example, they would further explain the social or technical context [42]. In addition, various family members appeared in the Zoom calls to support their parents or spouses [51]. This support sometimes hindered the instructions provided by the researchers. Finally, the “taking care of” was not only provided during the workshops but also continuously provided after the workshops were finished. The students continued to support several older adults as a way to further explore their needs concerning the use of digital devices and also to address their problems.

Fourth, care would not be possible if it were not accepted in the first place. That is why the phase of receiving care (“Care-receiving: valuing [evaluating and esteeming] of continued care acts”) is of key importance. Every action we performed within the PD was possible because of the acts of acceptance or rejection from the participants. The workshops were often very instructional or heavily moderator-oriented—the focus of each workshop was an activity that was presented by a moderator who provided the participants with instructions. Acceptance was then demonstrated by the participants following or trying to follow the instructions. However, sometimes they also rejected the instructions and either questioned the purpose of the activity (why should I do this?) or simply did not follow the instructions without further comments. Here, we were not sure if participants did not grasp the instructions (and did not feel competent or comfortable asking about them) or if they did not want to follow the instructions. In future meetings with research participants, we would need to determine ways to reflect on such matters.

Moreover, and in line with an interest in the present and future of pandemic technologies, during the COVID-19 pandemic, the last phase of care (“Caring with: care as a way to foster solidarity and trust among people”) especially emerged through our mutual collaboration with the researchers, instructors, and older adults. This meant not only that the older persons or that the university researchers cared, but that all participants also cared about the community in which they found themselves. The ACCESS project provided an institutional setting in which the participants could develop a disposition for care and practice care. The point was to take care of the project structure even during the pandemic so that a care network could be consolidated by providing learning opportunities.

The advantage of referring to the concept of “care” provided by Tronto [1,2] is that the needs of older adults are not understood as problems. It also makes it possible during the research process to reflect on how help should be provided and what practices can be considered appropriate [25,52].

Finally, part of our assumption of normality was that we, as researchers, should adopt a caring attitude toward older coresearchers because they are potentially more vulnerable than ourselves; however, the COVID-19 pandemic has disrupted many assumptions of normality. It has made older coresearchers’ needs for help even more visible, but it has also made us aware of our own vulnerabilities in terms of our research and everyday life. Furthermore, it has been shown that in some cases, coresearchers can deal better with the new situation [3]. Tronto’s [1,2] concept is also suitable for this because it fundamentally draws attention to the fact that the need for help and care is not limited to certain groups but that it is part of the human condition that each person, regardless of factors such as age or social status, has certain vulnerabilities that come into play in certain situations and result in a need for care and assistance.

### General Discussion

In principle, there are ethical requirements for all research, for example, that no people should be harmed or that personal data should only be used in anonymized form in scientific publications to protect privacy. In participatory research, that is, when academic researchers conduct research together with citizens, special ethical requirements and challenges arise, especially when dealing with susceptible groups, which may include older people. Considering this, it is no surprise that the researchers adopt a certain attitude of care for their study participants. However, using the ACCESS project as an example, care attitudes and practices can go well beyond what would generally be expected in the context of ethical PD.

Thus, the ACCESS project through its participatory approach opened a care “place” where it was possible to (1) engage with the older people from the project and foster their learning to use digital tools and (2) establish a care “place” as a window to understanding their daily issues that are heavily impacted by the COVID-19 restrictions or countermeasures with or without relationship to technology. Both aspects (engaging and understanding) are mutually elaborating, constituting, and fostering [31]*.* Connecting these findings to a broad understanding of care, through its PD approach focusing on digital literacy, the project became a way to *maintain participation* and personal relationships among the older participants and establish the demo kit website [41].

In the future, demographic changes will lead to an increased number of people in need of care. There will also be fewer people working in caring professions who can provide support. Therefore, it is important to consider how to address these challenges [53]. Our reflections learned from a crisis such as the COVID-19 pandemic provide a good lesson. Crises can also lead to discrimination and restrictions imposed on people based on their age, which can be reduced with appropriate sociotechnical measurements. If the use of videoconferencing tools led to people being able to talk to each other during lockdowns, it is also possible to assume that in future crises, these skills can be of benefit. Although the increased use of technologies also leads to a certain dependency, it is all the more important that people can use them in a self-determined manner.

The data from ACCESS have shown that it is relevant to focus on technologies as well as social practices. Health technologies enable individuals to take care of each other in different ways [54]. The ACCESS project addressed how people learn to use different technologies, where learning to use technologies is understood as a form of care [3,23]. People are enabled to interact with others without worrying about contracting COVID-19.

However, it is not enough to solely focus on the technologies and how they are used. It is also necessary to focus on the social structures in which help is available. Even if the state or families offer a lot of support, it is not enough in our view if only state actors or families perform the act of care in the future. It also requires suitable structures from the community in which people are located to be able to support each other. Various options must be available, including the use and appropriation of technologies to create this care place [55].

Our data show that many negotiations are still required regarding these considerations. Our view is that people in need should also have a say in how structures should be shaped or what technical artifacts should be used. However, such negotiation processes are often mediated by power structures that also address inequalities in care relationships. In care relationships, there is an unequal relationship between those who can provide care and those who receive it [26]. We tried to address these aspects using participatory methods and reflected on how care can be influenced by such aspects [27]. The participants need to see that they could contribute and also induce a positive change. Care is a practice that plays a role in all relationships and should be treated as a multifaceted contextual concept, involving discussion concerning how and for whom one should be caring. We suggest three aspects that support care: (1) soft skills, (2) attention and awareness, and (3) opportunities for performing the act of care.

### Soft Skills as Support for Care

In particular, when power imbalances exist, it is important for people to develop soft skills so that care can continue to be practiced. This also increases the motivation of the participants to continue performing the act of care. In the ACCESS project, people were given the opportunity to stay in touch, such as making small talk or exchanging pictures. These aspects should also be valued. This further shows that care is negotiated and that this process needs to be communicated. Older adults will accept the use of health technologies if it takes place in a pleasant atmosphere and they feel that their well-being is being addressed. This can also happen, for example, through the recognition of their learning progress and encouragement from others.

### Attention and Awareness as Support for Care

Digital tools and technology provide ways to attract people’s attention and an easy way for people to engage in conversations. Care also needs attention so that people can learn what to care about and in what ways to care. It is also about how care can be sustainable and how technical artifacts can support this [56].

### Opportunities for Doing Care as Support for Care

In our view, technologies offer opportunities to perform the act of care. Not only does it get people’s attention, but it is also possible to talk to each other about the technologies and find out what problems exist. Even if the people involved are heterogeneous, a common ground can still be found with reference to the technologies. It is mainly about being able to use the technologies and discover how they can be integrated into everyday life.

These 3 aspects have several practical implications for the adoption of care as a key principle in PD. First, on the web, it is important to understand the preexisting experiences of older adults with digital technologies (for example, by collecting information in advance about which devices and how many of them they own, how they are used to using them, or by establishing common vocabulary). Second, during the actual web-based engagement, it is important to develop the learning together and follow rules on how to behave in the web space, as well as place emphasis on verbal navigation between the different interfaces. Finally, in between web events, it is useful to promote interaction in an asynchronous mode, for example, through a messenger.

### Limitations

Research is never free of power dynamics, which can be found in various situations in participatory projects [57]. To give some examples, we were constantly working on how much we could adapt our scientific methods to the logic of the field. In our PD study, we tried to avoid being paternalistic, but it was rather challenging. We, as university researchers, were paid for what we did. The participants, by contrast, devoted their precious spare time to working with us [58]. The participants also had their own motivations and came voluntarily. One important aspect that should be emphasized more is that the researchers had a lot of support and help with the participants’ daily digital practices during the workshops, giving much “space” to acts of personal and individual counseling and help. We deliberately opened up the place of care because helping participants learn the mundane aspects of operating their tools enabled the researchers to gain more knowledge about everyday struggles with technology, participants’ sense-making processes, their interests, their learning paths, etc. These aspects are all interesting empirical elements of the negotiation of the research frame, research topics, methods chosen, and so forth. Negotiations were important, but participation is a permanent recognition, discussion, and constant attempt to find good balances in powerful moments. Therefore, the focus on care is also a way to strengthen the position of participants.

### Conclusions

Care is a broad concept that includes medical and physical care as well as help and support to promote mental and social well-being. In particular, during the COVID-19 pandemic, there was negotiation about who should care for others, how, and what practices might be considered helpful or harmful. In this paper, we examined how our research participants and we understood and promoted care. Through our exploration of PD during COVID-19, we demonstrated how care is an important basis for a genuine PD process and adopted an attitude of care to avoid any possibility of participants feeling excluded. Research participants should not be seen as a means to an end, and their well-being should be the main focus. An emphasis on establishing participatory care places can establish practices that help people individually. The ACCESS project originally aimed to develop interventions to foster digital literacy among older adults. The COVID-19 pandemic provided a very urgent real-world example, and the researchers broadened the design space and opened it up for care practices that were needed to shift the workshop settings from a physical setting to a remote, Zoom-based meeting place. However, there is also the need for older adults to acquire digital skills so that they could stay in touch with others during COVID-19. Thus, participatory research under pandemic conditions made visible the need for care as a pivotal part of participatory projects. This perspective helped to better understand the implications for the development and introduction of technologies that support older adults in their daily lives in a way they feel comfortable with but also better understand how technology needs to be embedded in a care place and combined with practices and strategies for learning and appropriation [59]. In this sense, the PD project during COVID-19 refers not only to caring for people but also integrating technologies into their everyday practices and empowering them to care for others. This aspect was also highlighted by de la Bellacasa [60]. Care, in this context, mediates both the intuitive (feeling) and rational (thinking) acts of older adults. With our analytical-conceptual, methodological, and empirical reflections, we would like to contribute to considering participation as a “joint accomplishment” under even crisis conditions and thereby consider how the dimensions of care can contribute to facilitating sociotechnical infrastructures for PD collaborations in the field of aging and welfare technologies [3]. PD as a care practice can be an enabler in this regard.

## Abbreviations

PD: Participatory Design
